# The BH3 mimetic ABT-737 increases treatment efficiency of paclitaxel against hepatoblastoma

**DOI:** 10.1186/1471-2407-11-362

**Published:** 2011-08-19

**Authors:** Justus Lieber, Carmen Eicher, Julia Wenz, Bettina Kirchner, Steven W Warmann, Jörg Fuchs, Sorin Armeanu-Ebinger

**Affiliations:** 1 Department of Paediatric Surgery and Paediatric Urology, University Children's Hospital, Hoppe-Seyler-Strasse 1, D-72076 Tübingen, Germany

## Abstract

**Background:**

The primary goal of current chemotherapy in hepatoblastoma (HB) is reduction of tumour volume and vitality to enable complete surgical resection and reduce risk of recurrence or metastatic disease. Drug resistance remains a major challenge for HB treatment. In some malignancies inhibition of anti-apoptotic pathways using small BH3 mimetic molecules like ABT-737 shows synergistic effects in combination with cystotoxic agents in vitro. Now we analysed toxicology and synergistic effects of this approach in HB cells and HB xenografts.

**Methods:**

Viability was monitored in HB cells (HUH6 and HepT1) and fibroblasts treated with paclitaxel, ABT-737 and a combination of both in a MTT assay. HUH6 xenotransplants in NOD/LtSz-scid IL2Rγnull mice (NSG) were treated accordingly. Tumour volume and body weight were monitored. Xenografted tumours were analysed by histology and immunohistochemistry (Ki-67 and TUNEL assay).

**Results:**

ABT-737 reduced viability in HUH6 and HepT1 cells cultures at concentrations above 1 μM and also enhanced the cytotoxic effect of paclitaxel when used in combination. Thereby paclitaxel could be reduced tenfold to achieve similar reduction of viability of tumour cells. In contrast no toxicity in fibroblasts was observed at the same regiments. Subcutaneous HB (HUH6) treated with paclitaxel (12 mg/kg body weight, n = 7) led to delayed tumour growth in the beginning of the experiment. However, tumour volume was similar to controls (n = 5) at day 25. Combination treatment with paclitaxel and ABT-737 (100 mg/kg, n = 8) revealed significantly 10 fold lower relative tumour volumes compared to control and paclitaxel groups. Paclitaxel dependent toxicity was observed in this mice strain.

**Conclusions:**

Our results demonstrate enhancement of chemotherapy by using modulators of apoptosis. Further analyses should include improved pharmacological formulations of paclitaxel and BH3 mimetics in order to reduce toxicological effects. Sensitising HB to apoptosis may also render resistant HB susceptible to established chemotherapy regimens.

## Background

Treatment results of children suffering from hepatoblastoma (HB) have been improved impressively during recent years. Current treatment strategies include neoadjuvant chemotherapy regimens and surgery in standard risk HB, achieving a 3-year overall survival probability of 96% [1-4]. However, the outcome of patients with high risk HB and relapsed HB (3-year survival: 69%) is still poor.

To improve outcome, therapy has been intensified using second-line cystostatic drugs in the standard treatment protocol for high risk HB [2]. Paclitaxel is mainly used in treatment regimes for ovarian, breast and non-small cell lung cancer. It has also been applied in paediatric patients with refractory malignancies and has been proposed as potential agent against high risk HB [5-9]. Paclitaxel stabilizes microtubules and as a result, interferes with the normal breakdown of microtubules during cell division [10]. This mitotic inhibitor promotes apoptosis as a secondary effect.

Apoptosis is an important factor in anticancer treatment and targeting this cell death mechanism has been constituted a promising alternative treatment option. HB cells express high amounts of anti-apoptotic molecules encoded by genes of the Bcl family [11,12]. Bcl-2, an important member of this family, blocks cytochrome C release by sequestering pro-apoptotic BH3-only proteins such as tBid, Bad, Bax, and Bim. Bcl-2 overexpression plays a central role in resistance to chemotherapy in multiple malignancies including HB [13]. Small BH3 mimetic molecules facilitate the activation of pro-apoptotic Bcl proteins by binding to the hydrophobic groove in Bcl-2 and Bcl-XL thus sensitizing tumour cells for apoptosis [14]. One of these molecules, ABT-737, was developed as an anti-tumour agent, induces apoptosis by selectively inhibiting the anti-apoptotic proteins Bcl-2, Bcl-XL, and Bcl-W. ABT-737 as a single agent has shown activity against several hematopoietic cell lines (leucaemias, multiple myeloma and cultured lymphoma) and some solid tumour cell lines, whereas efficiency was high in small cell lung carcinoma only [15-19]. Because ABT-737 does not block Mcl-1, it is anticipated that this drug will be most effective as a single agent against tumours that express low levels of these pro-survival protein [20]. Thus, ABT-737 may be active also in HB tumours, as gene expression analysis revealed a 2-fold lower expression of Mcl-1 in native HB tissue than in normal liver tissue [21]. Highly synergistic in vivo effects have been described when combining ABT-737 with established chemotherapeutic drugs [15,22]. In HB cells ABT-737 also induces apoptosis and enhances the effect of cytotoxic drugs (DOXO (doxorubicin), CDDP (cisplatin), paclitaxel and etoposide) commonly used in treatment protocols of HB [12].

In this study we describe the effects of ABT-737 in combination with paclitaxel in HB xenografts.

## Methods

### Drugs

ABT-737 and its enantiomer were kindly provided by Abbott (Abbott GmbH & Co. KG, Wiesbaden, Germany). For in vitro studies ABT-737 and its enantiomer were dissolved in DMSO at 1 mM and diluted with medium to a final concentration in the cell culture of 0.01, 0.1, 0.3, and 1 μM. For animal studies ABT-737 was slowly dissolved in a mixture consisting of 30% propylene glycol, 5% Tween80 and 65% dextrose in water. The vehicle was brought to a pH of 1.0 by adding 5 mol HCL to enable the compound to go into solution supported by sonication. After the compound was in solution, the pH was slowly raised to 3-4 by addition of aqueous NaOH. Paclitaxel was provided by Neocorp AG (Weilheim, Germany).

### Cells and culture conditions

The HB cell lines HepT1 [23] and HUH6 [24] were used for all experiments. The cells were transduced with a plasmid encoding gaussia luciferase (GLuc, pCMV-GLuc, NEB, Frankfurt am Main, Germany). Stable clones were isolated and maintained in DMEM (GIBCO BRL, Carlsbad, CA) supplemented with 10% FCS and G418. Tumour cells were grown as monolayer in Dulbecco's MEM medium (Biochrom, Berlin, Germany) supplemented with 10% fetal calf serum, 1% glutamine, and 2,5% HEPES buffer (Gibco, Eggenstein, Germany). The cells were grown at 37°C in a humidified atmosphere containing 5% carbon dioxide. All used cells were mycoplasma negative.

Fibroblasts were derived from human skin samples by tissue culture and grown as monolayer in the first three passages as described for tumour cells.

### Cell viability assay

HB cells (10,000 cells/100 μl) were seeded out in 96-well plates (Becton Dickinson GmbH, Heidelberg, Germany) and cultured as described above. At day two, paclitaxel was added to the cells at 7 different concentrations around IC_50 _[13]. ABT-737 was added to a final concentration in the cell culture of 0.01, 0.1, 1, and 3 μM. Experiments were repeated with fibroblasts and ABT-737 alone, as well as with ABT-737 combined with paclitaxel. Drug diluents were prepared shortly before administration. All assays were performed 3 times in quadruplicates.

Cell viability was assessed by MTT [3-(4.5-dimethylthiazol-2-yl)-2.5-diphenyl-tetrazoliumbromide]-assay (Sigma-Aldrich, Munich, Germany). 25 μl MTT (5 mg/ml) dissolved in PBS was added to each well. After incubation for 3 hours 100 μl/well lysis solution (10% SDS in acid water; Merck, Darmstadt, Germany) was added and further incubated over night in the dark at room temperature. Cell viability was assessed by measuring absorption at 570 nm using a Milena Kinetic Analyzer (DPC Bierman, Bad Nauheim, Germany). Percentages of cell viability were calculated by normalization of culture background without cells against untreated cultures as control. Dose dependent viability curves were computed by sigmoidal curves with variable slope to determine IC_50_.

### Apoptosis assay

For detecting apoptosis, the active caspase 3 assay was performed using Caspase-Glo™ 3/7 Assay according to the manufactory instructions (Promega, Mannheim, Germany). Briefly, 104 cells were seeded into a 96-well plate. After 24 h, the cells were treated with paclitaxel (0.1 and 0.3 μg/ml) and ABT-737 (0.3 μM). After treatment, the cells were harvested in Caspase substrate and luminescence was recorded for 10 seconds.

### Animals and Xenotransplantation

Xenotransplantation was performed as previously described [25]. All animal studies were approved by the local Government's ethical authority for animal experiments. (Regierungspräsidium Tübingen, Fachgebiet Tierschutz, Number K3/10). HUH6 cells were injected into the flank of 6-8 weeks old NOD/LtSz-scid IL2Rγnull mice (NSG). Animals were held under pathogen free conditions and were fed ad libitum with autoclaved food and sterilized water. For each tumour 0.2 ml of tumour cell suspension (2 × 10^6 ^cells) was injected subcutaneously into paravertebral areas. The observation time was 4 weeks, each group consisted of 6-8 animals. Treatment was initiated when tumours had reached a length of 5 mm. Paclitaxel in 200 μl saline solution was administered i.p. once per day on days 1-4 and 15-18 with a dosage of 12 mg/kg bodyweight. ABT-737 was administed i.p. with a dosage of 100 mg/kg bodyweight alone or in combination with paclitaxel using the same schedule. Drugs were prepared immediately prior to administration. Control animals were left untreated till day 25 unless tumour volume exceeded 1.5 cm^3^. Tumour volumes (V = 4/3π × a/2 × b/2 × c/2) and body weight of all animals were determined every 5 days. Relative tumour growth was calculated as a proportion of tumour volumes at each time point compared to day 0. Blood samples were taken from the retrobulbar plexus on days 0, 14, and 25. Serum GLuc activity was quantified in fresh serum. Therefore, 5 μl serum was added to 50 μl Gaussia GlowJuice (J.P.K. Instruments AG, Berlin, Germany) and GLuc activity was measured using a luminometer (Magic^® ^Lite Analysator, Ciba Corning) after adding 1 μl coelenterazine 100 μM to acquire photon counts for 10 sec. Activity was expressed as relative light units per second (RLU/s). Tumours were explanted on day 25 and prepared for histological analysis.

### Histology and Immunohistochemistry

Paraffin embedded sections obtained from xenografted tumours and liver were used for immunohistochemical staining against Ki-67. For each group 3 sections were cut (10 μm) from 4-5 different paraffined tumour blocks and mounted onto SuperFrost^® ^Plus microscope slides (R. Langenbrinck, Emmendingen, Germany). Sections were fixed and dehydrated using graded alcohol. The endogenous peroxidase activity was blocked by adding 0.03% Peroxidase (Merck, Darmstadt, Germany) for 10 min. Unspecific binding sites were blocked by incubation with phosphate buffered saline (PBS) containing 0.1% Tween 20 (PBST) (Sigma Aldrich, Munich, Germany) and 1% goat serum (Dako, Carpinteria, CA, USA) for 30 min. Sections were incubated with monoclonal mouse anti-human Ki-67 antibody (1:400; Dako) over night. Polyclonal rabbit anti-rat IgG antibody (1:100 biotin-labeled; Biozol, Eching, Germany) was used as secondary antibody. Avidin-biotin-peroxidase-complex (ABC) and DAB staining was applied using ABC-Kit PK-6100 standard (Linaris, Wertheim, Germany) according to manufacturer's protocol. Positively dividing cells were stained brown. For nuclear staining sections were counterstained in Mayer's Haemalaun solution for 30 sec. The proliferation index (PI) was calculated through division of the number of Ki-67 positive cell nuclei by the number of all tumour cells per high power field (20×). PI was given as mean of 3 randomly evaluated regions for all tumour samples. Sections were also stained using standard Mayer's Haemalaun as well as Eosin G-dilution and were analyzed by microscopy (Axioscope 40; Carl Zeiss, Oberkochen, Germany).

### TUNEL assay

Apotosis in explanted tumour tissue was assessed using TUNEL assay (Roche, Mannheim, Germany) according to the manufacturer's guidelines. Cells positive for apoptosis showed a green fluorescent signal and were visualized by fluorescence microscopy using a Zeiss Axio Scope epifluorescence microscope (Carl Zeiss GmbH, Oberkochen, Germany) and AxioVision software 3.1 (Carl Zeiss Vision, Aalen, Germany).

### Statistical analysis

Statistical analysis of relative tumour growth and body weights at distinct time points was carried out by one way ANOVA followed by Dunns' multiple test using GraphPad Prism 4.00 (GraphPad Software, San Diego, Califonia, USA, http://www.graphpad.com). Viability curves and tumour growth were fitted with a sigmoidal dose response function with variable slope. F-Test was used to compare curve parameters of treatment with and without ABT-737. All numeric data were expressed as means. Data plotted on graphs represent means and SD. Significance was assumed for all *p *< 0.05.

## Results

### Synergistic effect of paclitaxel and ABT-737 on HB cells

**Figure 1:**
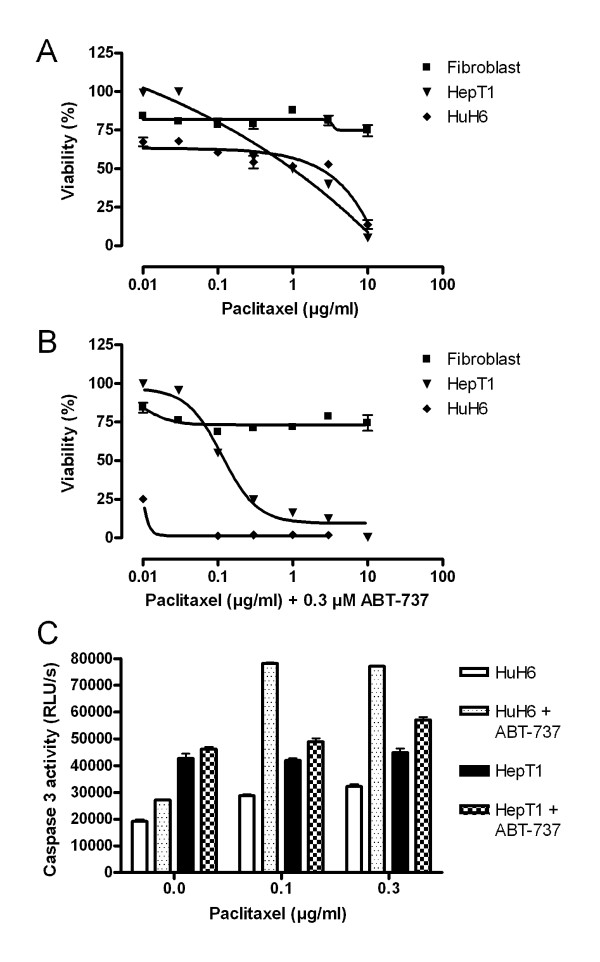
**Synergistic effect of paclitaxel and ABT-737 cultured cells**. Fibroblasts (black square), HepT1 (black triangle) and HuH6 (black rhomb) cells were incubated with paclitaxel at seven different concentrations alone (**A**) and in combination with 0.3 μM ABT-737 (**B**). Relative cell viability was determined 72 hrs later in a MTT assay. No toxicity was observed in fibroblasts. For HB cells synergistic effects of combined treatment could be observed. Apoptosis was detected by Caspase 3 activity 24 h after combination treatment of HuH6 and HepT1 cells (**C**). Enhanced Caspase 3 activity was found in HuH6 cells incubated with paclitaxel and 0.3 μM ABT-737 when compared with paclitaxel alone.

ABT-737 enhances the effect of various cytotoxic drugs in a combination treatment of tumour cell lines. To determine effects of treatment on HB cells a MTT assay was done. Paclitaxel alone led to a decreased viability in HepT1 and HUH6 cells (Figure 1A, B). IC_50 _of paclitaxel were 1 μg/ml in HepT1 cells and 6 μg/ml in HUH6 cells. Paclitaxel in combination with ABT-737 showed synergistic effects and considerable decrease of viability in HB cells. Combined paclitaxel (0.1 μg/ml)/ABT-737 (0.3 μM) treatment reduced cell viability in HepT1 cells to 50%. Treatment results were even more impressive in HUH6 cells. In this cell line only 25% of cells were vital after treatment with 0.01 μg/ml paclitaxel plus 0.3 μM ABT-737. The enantiomer of ABT-737 at 0.3 μM in combination with paclitaxel at 1 μg/ml did not reduce the viability of HB cells under 50% of a control (data not shown). In contrast, fibroblasts showed a dose-independent constant viability approximately 80% after treatment with paclitaxel alone and of approximately 75% after treatment with paclitaxel plus ABT-737. Paclitaxel induced apoptosis in HB cells as detected by quantifying Caspase 3 activity. In HUH6 cells a fourfold increase of Caspase 3 activity was detected when ABT-737 was added to paclitaxel. In HepT1 cells a higher background activity was observed compared to HuH6 cells and Caspase 3 activity was only slightly enhanced by paclitaxel or combination treatment (Figure 1C).

### Treatment of HB-xenografts with a combination of paclitaxel and ABT-737

**Figure 2:**
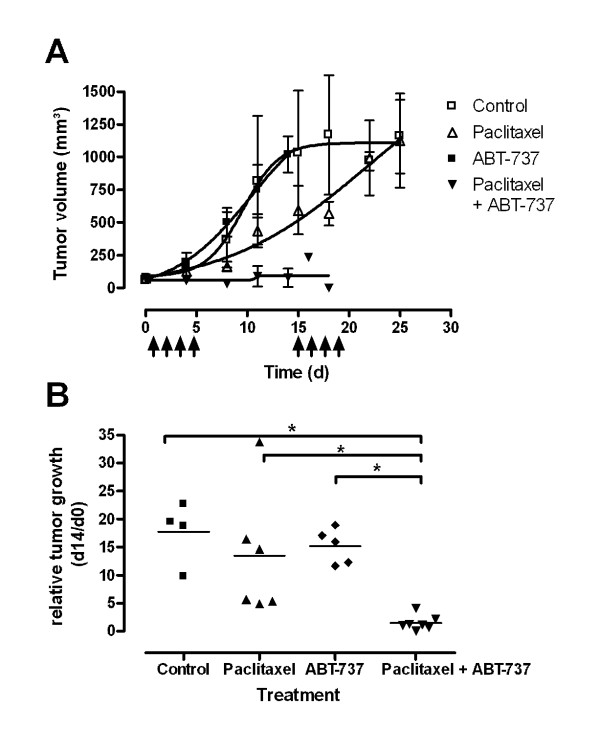
**Tumour growth after treatment with paclitaxel alone and in combination with ABT-737**. (**A**) HuH6 derived s.c. tumours were treated with paclitaxel (white triangle), ABT-737 (black square), and with a combination of both (black triangle) at indicated time points. Untreated animals served as controls (white square). Data points indicate means and SD of tumour volumes at respective days. (**B**) After the first cycle of treatment (14 days) the combination of paclitaxel and ABT-737 revealed a significantly lower relative tumour growth compared to controls and paclitaxel treated tumours. Data represent the relative tumour volume of each animal. *p < 0.05 in a Student t-test.

HUH6 xenografts were used to describe effects of ABT-737 in combination with paclitaxel in vivo. All xenotransplantated animals developed measurable tumours after 4-5 weeks. As an additional control parameter, expression of the transgene GLuc was detected in the blood of all mice at levels above 200 RLU/s, attesting tumour development. Tumour volume increased constantly in the control group (n = 5) (Figure 2A). A similar growth curve was observed in the group treated with ABT-737 alone (n = 5). Treatment with paclitaxel (n = 7) alone led to delayed tumour growth in the beginning of the experiment, but reached the mean tumour volume of the control group at day 25. Combined treatment of paclitaxel and ABT-737 (n = 8) did not show significant increase of tumour volume. In the first treatment cycle 3 animals of this group died. The remaining 5 animals died in the second cycle of treatment. Statistical analysis of tumour volumes between groups after completion of the first treatment course revealed significant lower relative tumour volume in the combined treatment group compared to control group and ABT-737 group (Figure 2B). Student t-test revealed a significant enhanced treatment efficiency of paclitaxel in combination with ABT-737 compared to paclitaxel alone (*p *= 0.01).

**Figure 3:**
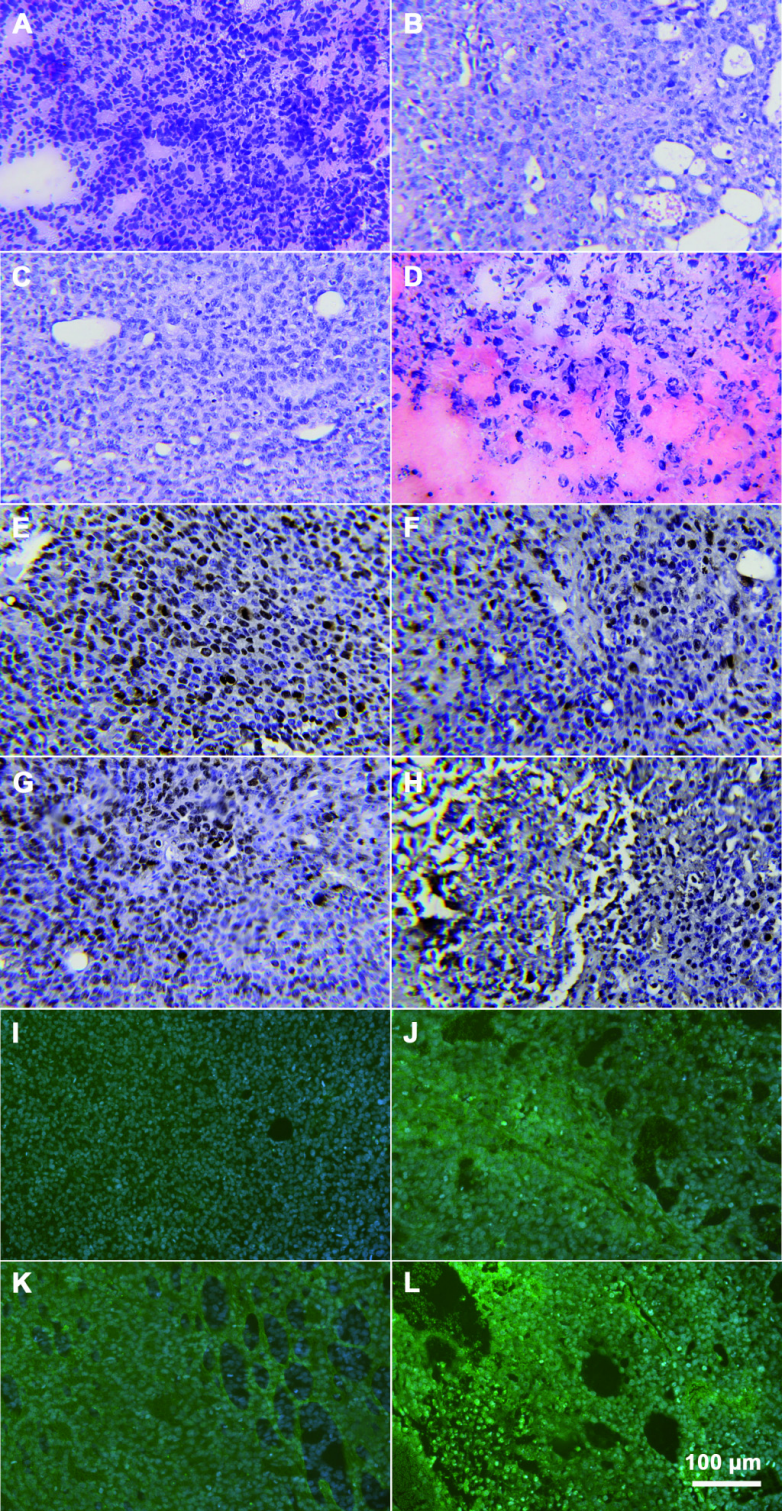
**Immunhistological analysis of HB tumours**. Images show staining of Haematoxilin and Eosin **(A-D)**, detection of Ki-67 **(E-H) **and Tunel assay **(I-L)**. For each representative samples of controls **(A, E, I)**, paclitaxel **(B, F, J)**, ABT-737 **(C, G, K) **and combination **(D, H, L) **are provided. Combined treatment using paclitaxel and ABT-737 reveals high tissue damage and a low proliferation index. Multiple picnotic nuclei denote necrotic tissue destruction. Brown staining shows Ki-67 positive cells. Bright green fluorescences are apoptotic cells. Nuclear staining was done by Hematoxylin and DAPI, respectively.

Immunhistological analysis of HB xenografts of the control group and paclitaxel group showed high density of tumour cells in HE staining (Figure 3). In contrast, multiple picnotic cells, hemorrhagic infarction, and large areas of necrosis were seen in the tumours of the combined treatment group. Brown staining of cells marked by anti-Ki-67 revealed dividing tumour cells. Cell proliferation was high in control tissues (PI 309 ± 109). Lower proliferation was observed in tumours treated with paclitaxel (PI = 233 ± 67) and ABT-737 (PI = 292 ± 38). Combination of both drugs further reduced proliferation (PI = 164 ± 21).

Apoptosis was detected by TUNEL assay. Thereby green fluorescent cells showed DNA fragmentation. The highest amount of apoptotic cells was seen in tumours after combined paclitaxel/ABT-737 treatment. Only in this group large areas of disintegrated tumours were detected. In summary, additive effects after paclitaxel and ABT-737 treatment were demonstrated in HB xenografts assessing tumour growth and histological appearance.

### Toxicity of paclitaxel in NSG mice

**Figure 4:**
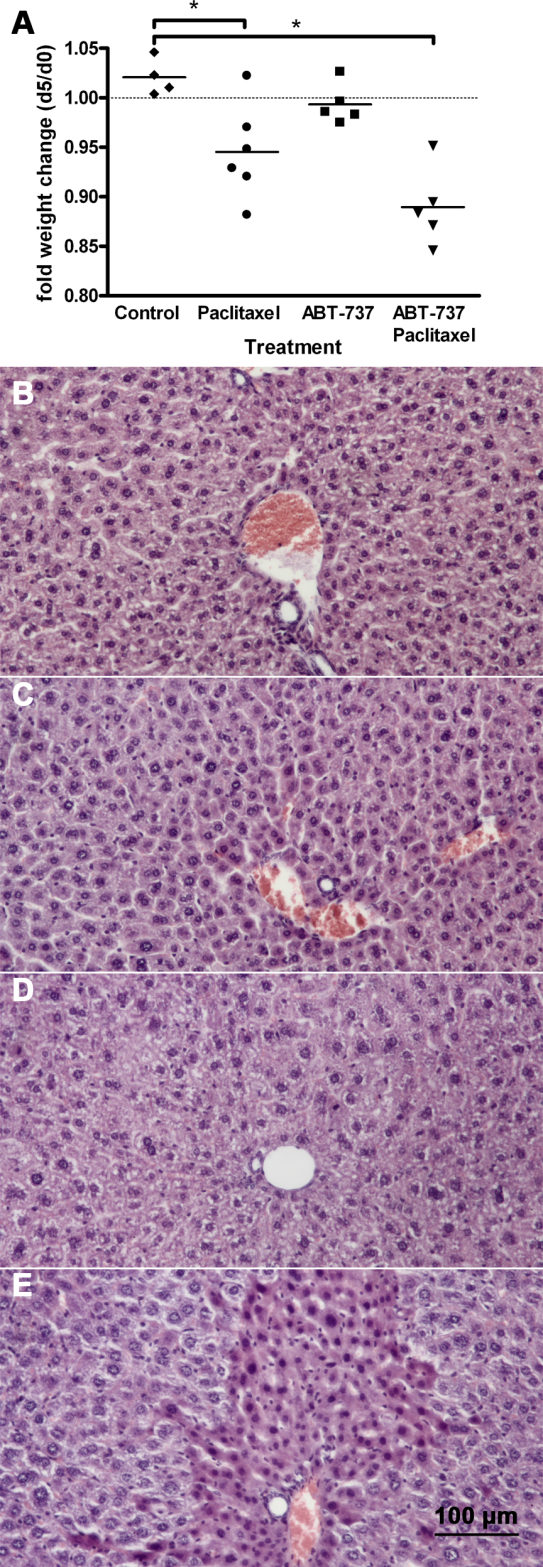
**Toxicity of paclitaxel in NSG mice**. NSG mice were treated as indicated on 4 consecutive days. The body weight is shown as fraction for each individual mouse at day 5 and at the beginning of the experiment (**A**). Significant loss of 10% body weight was observed in the groups treated with paclitaxel (black circle) and paclitaxel/ABT-737 (black triangle) compared with control (black rhomb) animals (one way Anova, Dunnett's multiple test p < 0.01). HE staining of liver tissues revealed multiple picnotic cells after combination treatment (**E**), which were not found in the other 3 groups: control (**B**), paclitaxel (**C**) and ABT-737 (**D**).

Toxicity was monitored by changes of body weight during treatment. Tumour bearing mice gained weight or remained constant during the 4 days of a treatment cycle (Figure 4A). Significant loss of 10% body weight was observed in the groups treated with paclitaxel or paclitaxel/ABT-737 compared with control animals (one way anova, *p *< 0.01). In the group treated with combined paclitaxel/ABT-737, 3 animals died in the first treatment cycle. The remaining 5 animals regenerated in the following 10 days, but died during the second cycle. Treatment with paclitaxel led to toxicity related death in 1 of 7 mice. Others showed general apathy. During necropsy no macroscopic tissue and organ changes were observed. No toxcitiy was observed after treatment with ABT-737 alone. Histological analysis of liver tissue after combined treatment with paclitaxel and ABT-737 detected islands of piknotic cell nuclei in HE staining at the timepoint of death (Figure 4B-E). In contrast, liver tissue of the control group and animals treated with paclitaxel or ABT-737 alone, did not show such changes of histological morphology at the end of the experiment (day 25).

## Discussion

Survival for children with HB is linked to complete resection of the primary tumour, which is possible in fewer than 50% of cases [1-3,26]. Chemotherapy plays an essential role in the treatment of HB by reducing extension of primarily unresectable tumours [1,2]. However, multi drug resistance develops in 80% of initially CDDP and DOXO sensitive patients after 4-5 courses of chemotherapy and remains a challenge in the optimization of treatment strategies. For high-risk and relapsed HB-characterized by complex drug resistance-various cytotoxic agents are used that have shown encouraging preclinical results as second line treatment in some interventional trials. Paclitaxel has proven efficiency in the treatment of HB cells in vitro and in xenotransplanted HB and has been proposed for the treatment of pediatric refractory malignancies [5,6,12]. In our study an initial tumour response to paclitaxel mono therapy was observed in xenografts. However, at the end of the observation time, tumours began to re-grow. Several mechanisms are currently under investigation in order to improve efficiency of chemotherapeutic agents [27].

One of them is modulation of apoptosis using small BH3 mimetic molecules, such as ABT-737, obatoclax, TW-37 and HA14 [14]. ABT-737 induces apoptosis as a single drug when treating various cell lines including HB in vitro and has previously shown additive effects when combined with various cytotoxic drugs including paclitaxel [12,15,17,22,28]. We observed additive effects of the combined therapy using paclitaxel and ABT-737 in a xenograft HB model, resulting in inhibition of tumour growth. Similar to our findings, CDDP reduced tumour growth when used alone but was more effective in some HB xenografts when combined with inhibitors of multi drug resistant proteins [25,29]. However, in this case the inhibitor targeted a putative induced expression of a particular drug resistant protein, which is also expressed in various normal tissues. In this study we used an inhibitor of an apoptosis modulating protein, thereby enhancing the tumour sensitivity to other drugs without compromising normal cells.

Paclitaxel is well tolerated in adults and children with some refractory or progressive solid tumours showing acceptable minor toxicity [30,31]. However, dose-dependent neurotoxicity and some local toxic effects such as abdominal pain after treatment with paclitaxel, which is rapidly cleared by the liver, have also been described [32,33]. In this study, toxicity of paclitaxel in NSG mice was observed. Other authors described no discernable increased toxicity, but used athymic NCr-nu ⁄ nu nude mice rather than NSG mice in their xenograft experiments [34,35]. The more resistant strains such as NMRI mice were omitted from these experiments because of a lower HB incidence after xenotransplantation [36].

Some pharmacological properties of paclitaxel were changed in previous studies by chemical derivation and liposomal formulation in order to reduce liver toxicity. Docetaxel, a semi-synthetic analogue of paclitaxel also leading to tumour regression, was described without significant toxicity in athymic NMRI mice [37]. The concept of reducing toxic effects by combining chemotherapeutics with natural compounds, such as beta-1,3-D-glucan, is very compelling, since some hepatotoxic side effects have finally been reported, even though higher paclitaxel dosage were used than in our study [38]. In addition, lyophilized paclitaxel magnetoliposomes demonstrated to be effectively delivered to the tumour and exert significant anticancer activity with fewer side effects when administrated parenterally in a xenograft mouse model for breast carcinoma [39]. However, we used paclitaxel in our study for a better comparability, since this agent had been used in previous studies of HB as well. Nevertheless, formulations with improved pharmacological properties might be more suitable for testing cytotoxic agents in NSG mice.

In contrast to paclitaxel, ABT-737 alone (100 mg/kg bodyweight) was not associated with toxic effects in this and other studies [15,22]. But when combining paclitaxel and ABT-737, toxicity was even higher than after treatment with paclitaxel alone. Treatment with paclitaxel led to toxicity related death in 1 of 7 mice, but in the group treated with combined paclitaxel/ABT-737 3 of 8 animals died in the first treatment cycle and the remaining 5 during the second. Histological analysis of liver tissue after combination treatment revealed multiple piknotic cell nuclei as a sign of induction of apoptosis also in healthy tissues instead of direct hepatic toxicity [40]. However, these local changes were unlikely to cause death exclusively. We assume that other tissues such as bowel and brain might have been affected, and will be assessed in further optimization studies. As the animals showed regeneration of body weight between the two cycles, elongation of the treatment free interval might reduce adverse side effects. Toxicity due to combination treatment may also be reduced by using second generation orally bioavailable BH3 mimetics, such as ABT-263 [41]. ABT-263 might further increase additive effects of combination treatment in HB cells in vivo compared to ABT-737. In this coherency, reduction of paclitaxel dosages might become usable with maintainance of anti-tumour activity and synchronous lowering of side effects.

## Conclusions

The primary goal of current chemotherapy in HB is reduction of tumour volume to enable complete surgical resection. Our results have proven optimization of chemotherapy by using modulators of apoptosis. However, improvement of pharmacological properties of both, paclitaxel and ABT-737, seems essential to reduce toxic side effects. Sensitising HB cells to apoptosis may restore sensitivity of resistant HB to established therapeutic regimens.

## Competing interests

The authors declare that they have no competing interests.

## Authors' contributions

CE and JW helped in animal experiments, JW and BK carried out the MTT assays and immunohistochemistry, SWW and JF helped to draft the manuscript, JL and SAE conceived of the experiments, participated in their design, performed the statistical analysis and coordination and drafted the manuscript. All authors read and approved the final manuscript.

## Pre-publication history

The pre-publication history for this paper can be accessed here:

http://www.biomedcentral.com/1471-2407/11/362/prepub
